# Zn-based seed priming enhances drought resistance during germination of *Lespedeza potaninii*: physiological and transcriptomic insights

**DOI:** 10.7717/peerj.21054

**Published:** 2026-04-07

**Authors:** Xiao wei Huo, Jian wei Li, Qi Chen, Yan Zhang, Rui Dai, Lin Bian, Na Guo, Qing wen Fu, Feng yan Yi, Zhi qiang Zhang

**Affiliations:** 1 Key Laboratory of Grassland Resources of the Ministry of Education, Key Laboratory of Forage Cultivation, Processing and Highly Efficient Utilization of Ministry of Agriculture and Rural Affairs, College of Grassland Science, Inner Mongolia Agricultural University, Hohhot, China; 2 Inner Mongolia Academy of Agricultural & Animal Husbandry Sciences, Hohhot, China

**Keywords:** Drought, *Lespedeza potaninii*, Nano, Transcriptomic

## Abstract

*Lespedeza potaninii* is a species of flowering plant in the legume family. Due to its strong stress tolerance and nitrogen-fixing capacity, *Lespedeza potaninii* has become a crucial plant species for reseeding and restoring degraded grasslands in China. However, drought stress during the seedling or germination stage severely impacts the effectiveness of *Lespedeza potaninii*-based grassland restoration efforts. To screen seed-soaking agents enhancing seed germination under sorbitol-simulated drought stress and explore the underlying mechanisms, seed germination, physiological parameters and transcriptomic regulation of *Lespedeza potaninii* seeds under different seed-soaking treatments were analyzed. The results revealed that sorbitol-simulated drought stress significantly reduced seed germination of *Lespedeza potaninii*. However, treatments with 0.04% ZnSO_4_ or 0.10 mg/L ZnO-NPs could significantly enhance germination percentages. 0.04% ZnSO_4_ and 0.10 mg/L ZnO-NPs treatments notably decreased hydrogen peroxide (H_2_O_2_) levels, with 0.10 mg/L ZnO-NPs also increasing glutathione cycle efficiency. Transcriptomic analysis revealed that a 0.10 mg/L ZnO-NPs treatment improved drought resistance by enhancing energy and nutrient metabolism, particularly nitrogen metabolism, which in turn strengthened antioxidant defenses through the glutathione metabolism. In contrast, the ZnSO_4_ treatment promoted sulfur metabolism, thereby enhancing glutathione metabolism and increasing the biosynthesis of flavonoids and monoterpenes, further improving drought resistance. Both treatments likely involve zinc ions in promoting antioxidant synthesis and maintaining cell membrane stability by regulating antioxidant defense, particularly glutathione metabolism. These findings offer novel insights and strategies for improving seed emergence and establishment in degraded grasslands.

*Lespedeza potaninii* is a native perennial legume of desert grasslands and steppe-desert regions, notable for its strong drought and salt tolerance (Chen et al., 2024). It therefore plays a key ecological role and is important for grassland restoration. However, seed germination and seedling establishment are often impeded by soil drought resulting from ecological degradation (Kimura et al., 2022; Bardgett Richard et al., 2021). This limitation poses a major obstacle to effective restoration, particularly in areas experiencing persistent degradation (Aoyama, Cook & Hallett, 2023).

Drought stress strongly limits plant growth and development, and seed germination is especially vulnerable because it depends on water availability (Zhu et al., 2024). Reduced water uptake impairs the cellular processes required for germination and thus delays early seedling establishment (Lu et al., 2022). To cope with drought, seeds activate antioxidant enzymes and modulate drought-responsive genes, phytohormones, and metabolic pathways (Bhattacharjee et al., 2023; Garstecka et al., 2023; Huang et al., 2023a). For example, dandelion (*Taraxacum mongolicum*) seeds exposed to drought show elevated activities of superoxide dismutase (SOD) and peroxidase (POD) and increased levels of osmolytes such as soluble sugars, malondialdehyde (MDA), and Pro (Wu et al., 2022). In soybean (*Glycine max*), drought disrupts sucrose metabolism and its balance in leaves and seeds during reproduction, which reduces seed weight (Du et al., 2020). A genome-wide association study of 165 rice (*Oryza sativa*) accessions found that *OsGA2ox5* improves drought tolerance during seed germination by regulating carbohydrate metabolism (Yang et al., 2024). Under drought stress, soaking rice seeds in 100 μM melatonin markedly increases germination percentage and seedling biomass (Li et al., 2022). Likewise, melatonin treatment improves soybean seed germination under drought, stabilizes cell membranes at 500 μmol/L, and raises activities of antioxidant enzymes including SOD, POD, Catalase (CAT), and ascorbate peroxidase (APX) (Zhang et al., 2020a). Exogenous indole-3-acetic acid (IAA) enhances drought tolerance in white clover (*Trifolium repens* L.) by activating genes associated with auxin, abscisic acid, and jasmonic acid signaling while repressing senescence-related genes (Zhang et al., 2020b). In addition, diethyl aminoethyl hexanoate (DA-6) increases drought resistance in pineapple (*Ananas comosus*) by elevating protease activity and overall antioxidant capacity (Huang et al., 2023b).

Nanoparticles have recently become a major focus of scientific research. They can modulate plant growth and development, break seed dormancy, and regulate nutrient metabolism and uptake, thereby affecting the entire growth process (Wang et al., 2024). For example, coating fragrant rice seeds with iron oxide nanoparticles significantly enhances germination and alters early growth and physiological parameters (Lin et al., 2024). Similarly, exogenous silicon dioxide nanoparticles (SiNPs) substantially improve physicochemical conditions, yield components, and seed nutritional quality of legumes under salt stress (Sarkar, Sarkar & Roy, 2024). Seed soaking and foliar application of iron nanoparticles (FeNPs) also produce stronger benefits for alfalfa growth and nitrogen fixation than FeCl_2_, while promoting competitive and cooperative interactions among rhizosphere fungal communities (MingXu et al., 2023).

Metal-based nanoparticles reduce drought-induced oxidative damage by lowering reactive oxygen species such as H_2_O_2_ and O^2−^. They also improve osmotic regulation during drought by raising levels of osmolytes and osmotic regulators, including proline, glycine betaine, soluble sugars, and amino acids (Mustafa et al., 2021; Van Nguyen et al., 2022). Zinc (Zn), an essential micronutrient, plays a central role in enhancing drought resistance through multiple physiological and molecular mechanisms (Umair Hassan et al., 2020). Under drought, zinc supplementation improves seed germination, plant water relations, cell membrane stability, osmotic adjustment, stomatal behavior, water use efficiency Water Use Efficiency (WUE), and photosynthesis, which collectively boost plant performance (Muhammad et al., 2022). Zinc also alleviates drought damage by increasing photosynthetic rate and pollen viability and, in particular, by improving WUE, thereby raising wheat (*Triticum aestivum* L.) yield (Karim et al., 2012). Jing et al. (2022) showed that Zn^2+^ enhances resistance in tobacco (*Nicotiana tabacum* L.) by activating signal transduction and modulating resistance-related gene expression. Under Zn^2+^ treatment, membrane transport proteins direct zinc to epidermal cells, and transcription factors, notably zinc-finger proteins, regulate growth and responses to biotic and abiotic stress (Mapodzeke et al., 2021). Maintaining adequate Zn^2+^ concentrations therefore substantially enhances plant stress resistance.

Nano zinc oxide (ZnO) exhibits potent antibacterial properties and can promote seed germination and plant growth (Abhilash et al., 2023). For example, ZnO-NPs substantially increased the germination percentage and vitality index of onion (*Allium cepa* L.) (Rajesh et al., 2024). Tomato (*Solanum lycopersicum* L.) seeds treated with 300 and 400 µg/mL ZnO-NPs showed higher germination percentages and enhanced seedling growth (Duraisamy et al., 2022). Montanha et al. (2020) found that applying zinc oxide nano-coatings (4 mg/g) to soybean seeds resulted in zinc uptake during germination and seedling growth; residual zinc then altered rhizosphere soil pH, creating more favorable conditions for seedling development and improving soybean germination. Conventional zinc sources have also been studied extensively. ZnSO_4_ application increased protein content and nutritional quality in peanut (*Arachis hypogaea* L.), and ZnSO_4_ seed soaking significantly improved germination percentage in quinoa (*Chenopodium quinoa* Willd.) (Bourhim et al., 2022; Lal et al., 2023; Reddy, Mehera & Kumar, 2023).

We evaluated seed-soaking agents that might improve *Lespedeza potaninii* germination under sorbitol-simulated drought stress by comparing distilled water (CK), ZnSO_4_, and ZnO-NPs. To elucidate how ZnSO₄ and ZnO-NPs affect germination, we measured physiological indicators and analyzed transcriptomic profiles of seeds treated with these agents. These results will provide technical guidance and theoretical understanding for applying *Lespedeza potaninii* in drought-prone environments.

## Materials and Methods

### Breaking the seed hardiness of *Lespedeza potaninii*

(1) Sandpaper treatment: Seeds were subjected to repetitive rubbing with sandpaper until visible abrasions appeared on their surfaces. Following the sandpaper treatment, the seeds were immersed in water for 24 h. Each treatment was replicated three times, with the control group (CK) remaining untreated.
(2) Concentrated sulfuric acid treatment: A specified quantity of seeds was placed in a container with 10 mL of 98% concentrated sulfuric acid and gently stirred with a glass rod to ensure complete immersion, with exposure times of 10, 20, and 30 min. Post-treatment, the seeds were rinsed under tap water for 3 min and then soaked in water for 24 h. Each treatment was replicated three times, with the control group (CK) remaining untreated.
(3) Hot water treatment: A specified quantity of seeds was placed in water heated to 110 °C, allowed to cool naturally, and then soaked in water for 24 h. Each treatment was replicated three times, with the control group (CK) remaining untreated.
(4) Liquid nitrogen treatment: A specified quantity of seeds was placed in a container with liquid nitrogen for 2 and 5 min, respectively, and then immediately transferred to a 30 °C water bath for 10 min. Post-treatment, the seeds were rinsed under tap water for 3 min and soaked in water for 24 h. Each treatment was replicated three times, with the control group (CK) remaining untreated.
(5) NaClO: Seeds were immersed in a 98% analytical grade sodium hypochlorite solution for 10 min, rinsed under tap water for 3 min, and then soaked in water for 24 h. Each treatment was replicated three times, with the control group (CK) remaining untreated.

### Characterization of ZnO-NPs

ZnO-NPs (30 ± 10 nm, density 5.61 g/cm^3^, purity 99.9%) were obtained from Shanghai Macklin Biochemical Co., Ltd. (Shanghai, China). Scanning electron microscopy (Gemini SEM 300; ZEISS, Oberkochen, Germany) was used to characterize particle size and morphology. Energy-dispersive spectroscopy (EDS) determined elemental composition. Figure S1 presents the nanoparticle size and the distribution of the principal elements (Zn, O), confirming the identity and characteristics of the ZnO-NPs.

### Seed soaking

*Lespedeza potaninii* seeds (collected in 2022 in Inner Mongolia Autonomous Region, China) treated with concentrated sulfuric acid for 30 min were selected as the experimental material. The seeds were subsequently soaked in the following solutions: 0.01%, 0.04%, 0.06%, and 0.10% ZnSO_4_; 0.01, 0.05, 0.10, 0.20, and 0.40 mg/L ZnO-NPs. All treatments involved a 12-h soaking period, with seeds soaked in water serving as the control (CK). During the soaking process, solutions were maintained at a constant temperature with continuous stirring. The concentration of the seed soaking agent that yielded the highest seed vitality index was selected for subsequent experiments. For sorbitol-simulated drought stress experiments, seed soaking agents were sterilized, and the soaking process was conducted in a laminar flow clean bench to ensure a sterile environment.

### Simulated drought stress

*Lespedeza potaninii* seeds treated with a seed soaking agent were selected as the experimental material. Sorbitol was used as the drought-inducing agent to prepare the stress media. Four drought gradient treatments were applied: 0, 0.10, 0.24, and 0.48 mol/L sorbitol, representing CK, mild drought stress, moderate drought stress, and severe drought stress, respectively. The pH was adjusted to 5.8, and the media were sterilized at 121 °C, then cooled to 50 °C before use. Each treatment was conducted for a specified duration under controlled conditions to ensure accuracy and replicates (Table 1).

**Table 1:** Drought-simulated culture medium formulation.

Drought reagent	CK	0.1 mol/L mild drought.	0.24 mol/L moderate drought.	0.48 mol/L severe drought.
MS	4.74 g/L	4.74 g/L	4.74 g/L	4.74 g/L
Sucrose	20.00 g/L	20.00 g/L	20.00 g/L	20.00 g/L
Agar	6.00 g/L	6.00 g/L	6.00 g/L	6.00 g/L
Sorbitol	0	18.217 g/L	43.7208 g/L	87.4417 g/L

**Note:**

MS, Murashige and Skoog Medium.

### Germination test and measurements

**(1) Germination rate of *Lespedeza potaninii* under different seed hardiness breaking treatments**

A total of 100 seeds from each treatment of *Lespedeza potaninii* were selected for germination using the Petri dish paper method (TP method). Seeds were placed on Petri dishes lined with two layers of filter paper and subjected to 12 h of light at 25 °C followed by 12 h of darkness at 20 °C, over a 7-day germination period, with three replicates per treatment. Germination potential was assessed at peak germination, the germination percentage was calculated after 7 days, and shoot lengths were measured.

**(2) Drought tolerance evaluation**

A total of 100 treated seeds were germinated in a culture medium under similar conditions with three replicates. Due to simulated drought stress in the culture medium, germination and growth differed from standard conditions, resulting in slower germination, reduced growth rates, and a longer germination period. Based on observed trends, the germination period under sorbitol-simulated drought stress was extended to 15 days.

### Germination test

Based on the recorded daily germination counts, the germination percentage (GR), germination energy (GE), germination index (GI), and vitality index (VI) of *Lespedeza potaninii* were calculated using the following formulas: Number of seeds tested; Number of normal seedlings at peak.

(1) $${\rm GR} = {\rm Number\; of\; germinated\; seeds}/{\rm Total\; Number\; of\; seeds\; tested} \times 100 \rm \%.$$

(2) $$\mathrm{GE = Number\, of\, normal \,seedlings\, at\, peak/ Total\, number \,of\, seeds\, tested\times100\% }.$$

(3) $${\rm GI} = { \Sigma }\left( {\displaystyle{{{\rm Gt}} \over {{\rm DT}}}} \right).$$

(4) $${\rm VI} = {\rm GI} \times {\rm S\; }.$$

Note: Gt is the number of germinations on day t, Dt is the corresponding number of germination days, and S is the root length of the seedling.

### Determination of physiological indices

*Lespedeza potaninii* seeds, pre-soaked in 0.10 mg/L ZnO-NPs, 0.04% ZnSO_4_ or distilled water (CK), then quickly frozen in liquid nitrogen and preserved at −80 °C in an ultra-low temperature freezer for subsequent physiological measurements and transcriptomic analysis. The contents of SOD, POD, CAT, soluble sugars, glutathione peroxidase (GSH-px/GPX), glutathione reductase (GR), oxidized glutathione (GSSG), reduced glutathione (GSH), hydrogen peroxide (H_2_O_2_), and MDA were measured. The physiological indices were assessed using reagent kits, all procured from Solarbio Science & Technology Co., Ltd., Beijing, China.

### Transcriptomic analysis

#### RNA preparation, cDNA library construction, and RNA sequencing

Transcriptomic sequencing was conducted on *Lespedeza potaninii* seeds subjected to treatments of 0.10 mg/L ZnO-NPs, 0.04% ZnSO4, and distilled water (CK), each with three biological replicates. The samples were promptly frozen in liquid nitrogen and stored at −80 °C. Total RNA was extracted using the MJZol kit, and its concentration and purity were evaluated using a Nanodrop 2000. RNA integrity was verified through agarose gel electrophoresis. mRNA was purified using Oligo(dT) magnetic beads, fragmented, and utilized for cDNA synthesis. Sequencing was carried out on an Illumina NovaSeq 6000 platform by Shanghai Majorbio Bio-Pharm Technology Co., Ltd. The raw sequences can be accessed in the NCBI database under accession number PRJNA1095388. The experimental design yielded a statistical power of 0.9870239, as calculated by RNA Seq Power. To ensure data quality and reliability, raw reads were filtered to produce high-quality sequencing data (clean data). After quality control, these clean reads were aligned to the reference genome. Gene and transcript expression levels were then quantified using RSEM software (Shuang et al., 2025).

### Differential expression genes (DEGs) analysis

For projects with multiple samples (≥2), differential expression analysis was performed on the gene read counts to identify differentially expressed genes (DEGs). DESeq2 was used for these analyses. Genes were considered significantly differentially expressed under the default criteria: FDR < 0.05 and |log2FC| ≥ 1. Identified DEGs were annotated against the Gene Ontology (GO) (http://geneontology.org/) and Kyoto Encyclopedia of Genes and Genomes (KEGG) (https://www.genome.jp/kegg/) databases. GO enrichment analysis was conducted with Goatools using Fisher’s exact test. To limit false positives, *P*-values were adjusted by four multiple-testing correction methods: BH, BY, Holm, and Bonferroni. Software utilized: Goatools (https://github.com/tanghaibao/GOatools). KEGG pathway enrichment analysis was performed with the Python scipy package, using the same principles applied to GO functional enrichment. After obtaining gene read counts, differential expression analysis was performed on projects with multiple samples (≥2) using DESeq2. Significantly differentially expressed genes were defined by FDR < 0.05 and |log2FC| ≥ 1. Gene annotation used the Gene Ontology (GO) and Kyoto Encyclopedia of Genes and Genomes (KEGG) databases. GO enrichment analysis was carried out with Goatools and Fisher’s exact test. *P*-values were adjusted with multiple-testing corrections (BH, BY, Holm, Bonferroni) to control false positives. Goatools was used for these adjustments. KEGG pathway enrichment analysis was conducted with the Python scipy package according to the same principles as the GO analysis.

### Statistical analysis

Collected data were organized and analyzed in Microsoft Excel 2019. For statistical validation, one-way analysis of variance (ANOVA) was performed in SPSS Statistics 27.0. Results were plotted in Origin 2021 to visualize trends and patterns.

## Results

### Seed hardiness breaking

**Figure 1:**
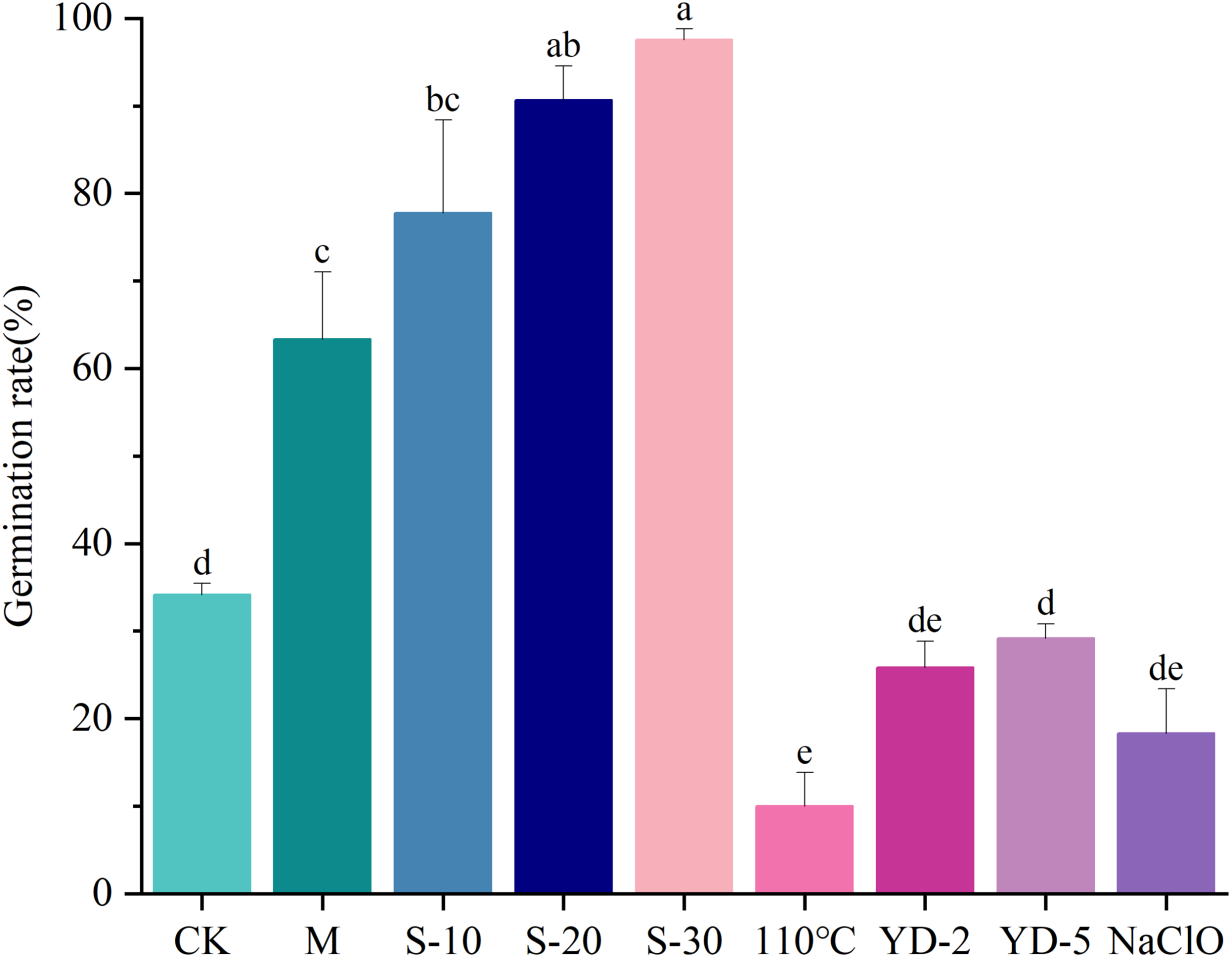
Effects of different seed hardiness breaking treatments on the germination percentage of *Lespedeza potaninii*. Note: CK denotes untreated *Lespedeza potaninii* seeds; M denotes seeds subjected to sandpaper abrasion; S-10, S-20, and S-30 denote seeds soaked in concentrated sulfuric acid for 10, 20, and 30 min, respectively; 100 °C denotes seeds soaked in water at 100 °C; YD-2 and YD-5 denote seeds soaked in liquid nitrogen for 2 and 5 min, respectively; and NaClO denotes seeds soaked in sodium hypochlorite.

After applying various dormancy-breaking treatments to *Lespedeza potaninii* seeds, sandpaper abrasion and sulfuric acid soaking were found to substantially increase germination, thereby overcoming seed hardiness. A 30-min sulfuric acid soak produced the highest germination rate, 97.56%, which significantly exceeded the rates achieved by the other treatments (Fig. 1).

### Effects of different seed soaking agents on germination ability of *Lespedeza potaninii*

**Figure 2:**
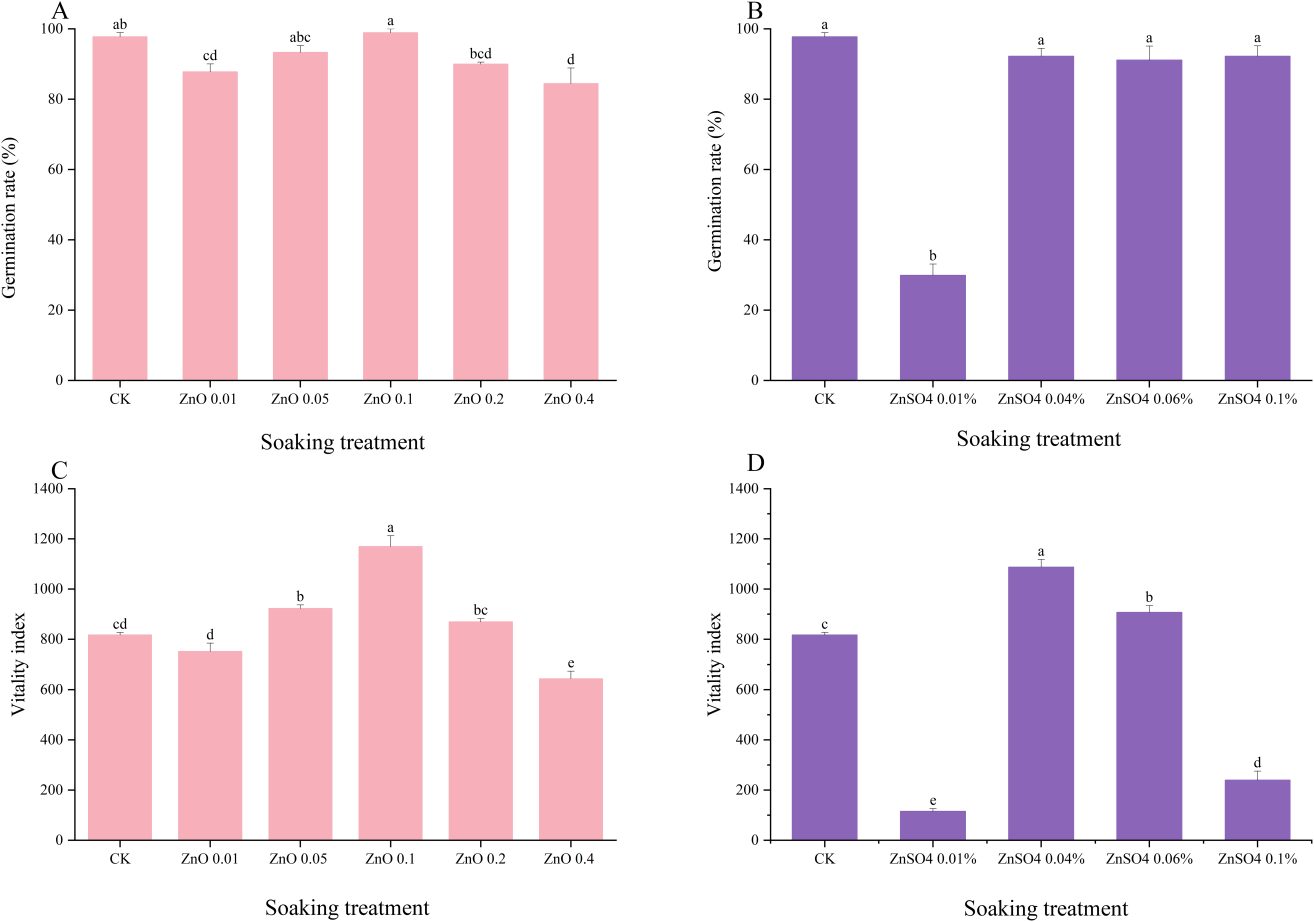
Effects of different concentrations of seed soaking agents on the germination indicators of *Lespedeza potaninii*.

Pre-sowing seed soaking is a common practice in agriculture that improves seed viability, accelerates germination, increases germination rates, and enhances stress resistance (Sharaf-Eldin et al., 2022; Tang et al., 2022). In *Lespedeza potaninii*, seed germination was not affected by any soaking treatment except 0.01% ZnSO_4_, which significantly inhibited germination. Soaking agents at different concentrations produced distinct effects on the seeds’ vitality index. Specifically, 0.10 mg/L ZnO-NPs and 0.04% ZnSO4 treatments significantly increased the vitality index compared with other concentrations and the CK (Fig. 2).

### Effects of different seed soaking agents on germination ability of *Lespedeza potaninii* under simulated drought stress

**Figure 3:**
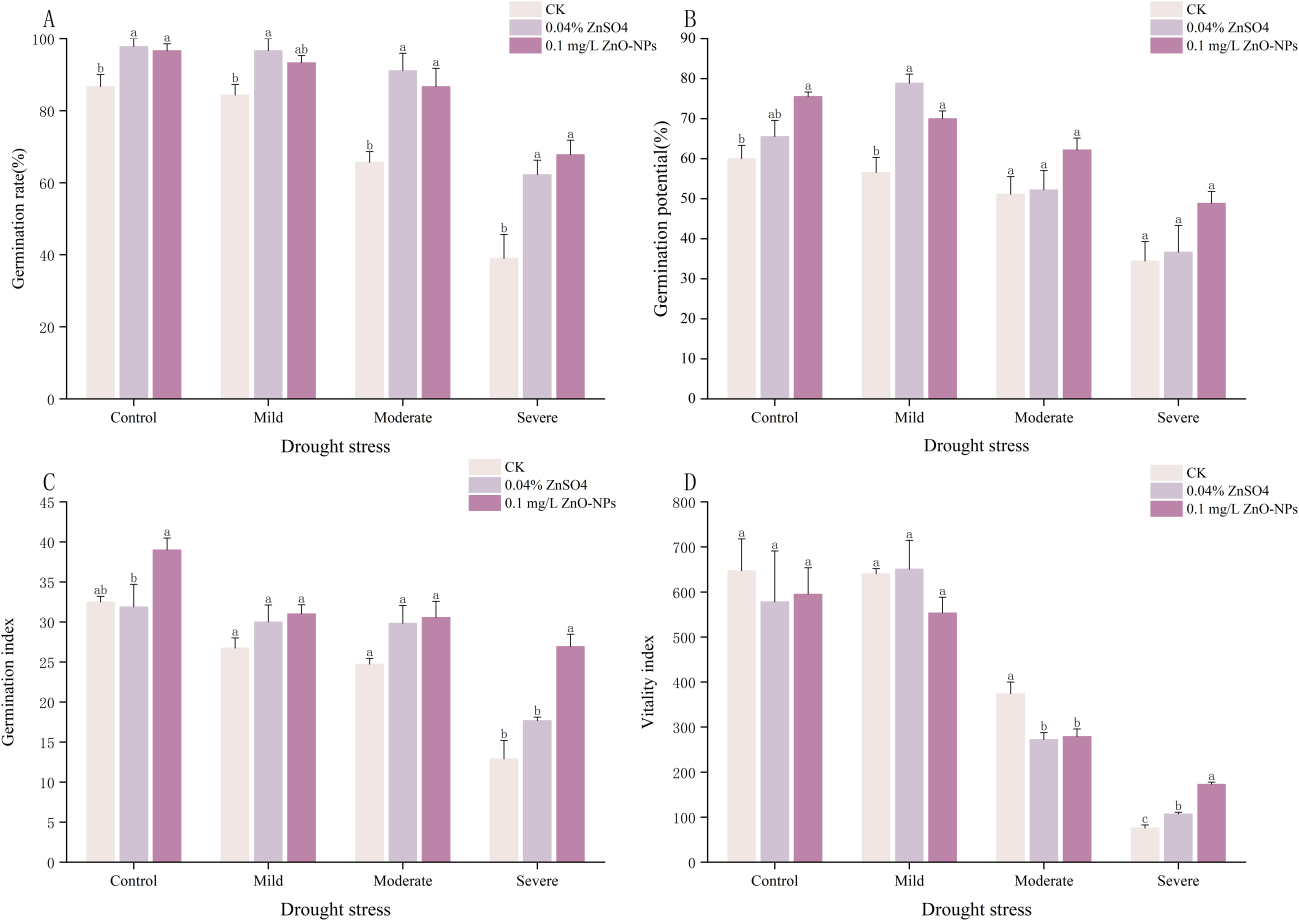
(A–D) Effects of different seed soaking agents on germination indices of *Lespedeza potaninii* under drought stress.

Under non-drought conditions, germination percentages of *Lespedeza potaninii* seeds treated with various soaking agents did not differ significantly from the control (CK). In contrast, under moderate and severe simulated drought stress, seeds soaked in 0.04% ZnSO_4_ showed germination percentages 25.44% and 23.22% higher than CK, respectively. Specifically, under severe simulated drought stress, seeds treated with 0.10 mg/L ZnO-NPs exhibited a significant 28.78% increase in germination percentage relative to CK (*P* < 0.05). After mild simulated drought stress, seeds treated with 0.10 mg/L ZnO-NPs and 0.04% ZnSO_4_ had significantly higher germination potential than the other treatments, increasing by 23.78% and 39.5% over CK, respectively (*P* < 0.05, Figs. 3A, 3B). Under severe simulated drought, the germination index of ZnO-NPs–treated seeds was significantly greater than CK, but did not differ significantly from the 0.04% ZnSO4 treatment (*P* < 0.05, Fig. 3C). Finally, under moderate and severe simulated drought stress, seeds treated with 0.10 mg/L ZnO-NPs had a significantly higher vitality index than the other treatments; under severe drought, the 0.04% ZnSO_4_ treatment also produced a vitality index significantly higher than CK (*P* < 0.05, Figs. 3D, S2–S4).

### Effects of different seed soaking agents on physiological indices of *Lespedeza potaninii*

**Figure 4:**
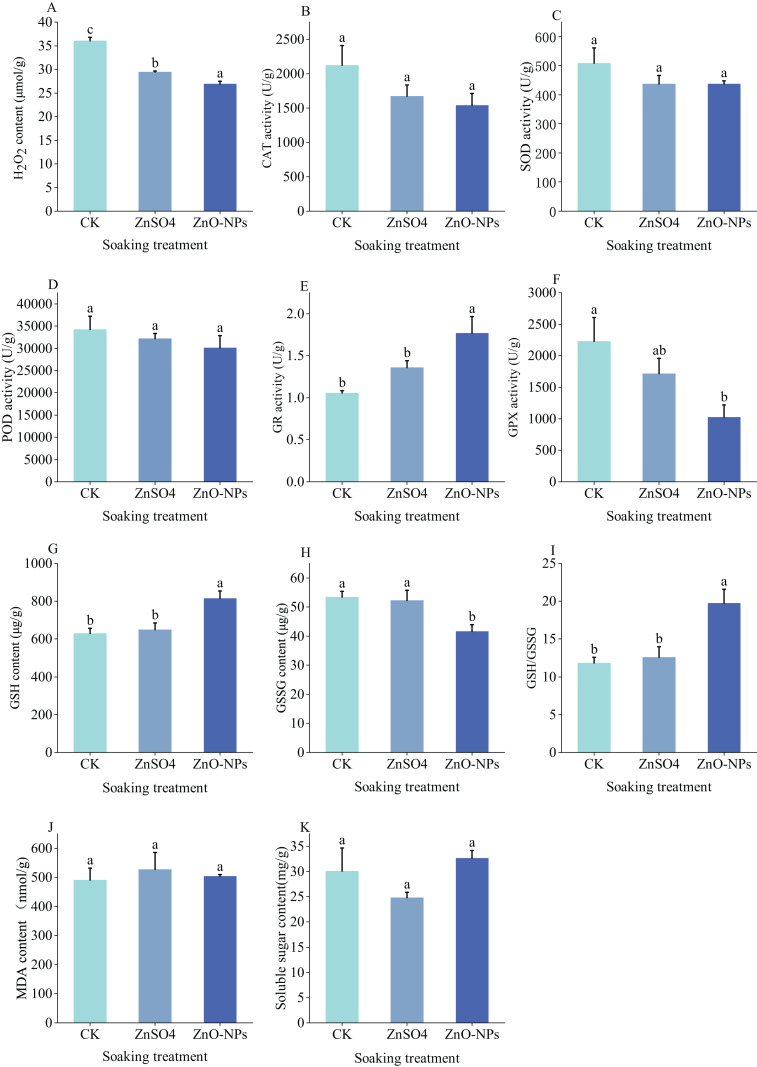
(A–K) Effects of different seed soaking agents on oxidase, secondary metabolites and osmotic adjustment substances of *Lespedeza potaninii*.

Plants deploy multiple antioxidant systems to mitigate reactive oxygen species (ROS) damage during stress, thereby maintaining intracellular redox balance, preserving membrane integrity, and supporting normal growth and development. Seed soaking with 0.04% ZnSO_4_ or 0.10 mg/L ZnO-NPs significantly reduced H_2_O_2_ content relative to CK (*P* < 0.05, Fig. 4A). In contrast, CAT and SOD activities, MDA, and soluble sugar content in *Lespedeza potaninii* seeds did not differ significantly across the seed-soaking treatments (Figs. 4B, 4C, 4D, 4K and 4L). Glutathione metabolism is critical for sustaining a reducing environment in plants and for scavenging ROS. Notably, 0.10 mg/L ZnO-NPs markedly altered enzymes in this pathway. Compared with CK, this treatment significantly increased GR activity and decreased GSH content, while GPX activity and GSSG content were significantly lower than CK (*P* < 0.05, Figs. 4E–4I). Consequently, the GSH/GSSG ratio in seeds treated with 0.10 mg/L ZnO-NPs was significantly higher than in CK, with an increase of 7.92 (*P* < 0.05, Fig. 4I).

### Transcriptomic analysis

**Figure 5:**
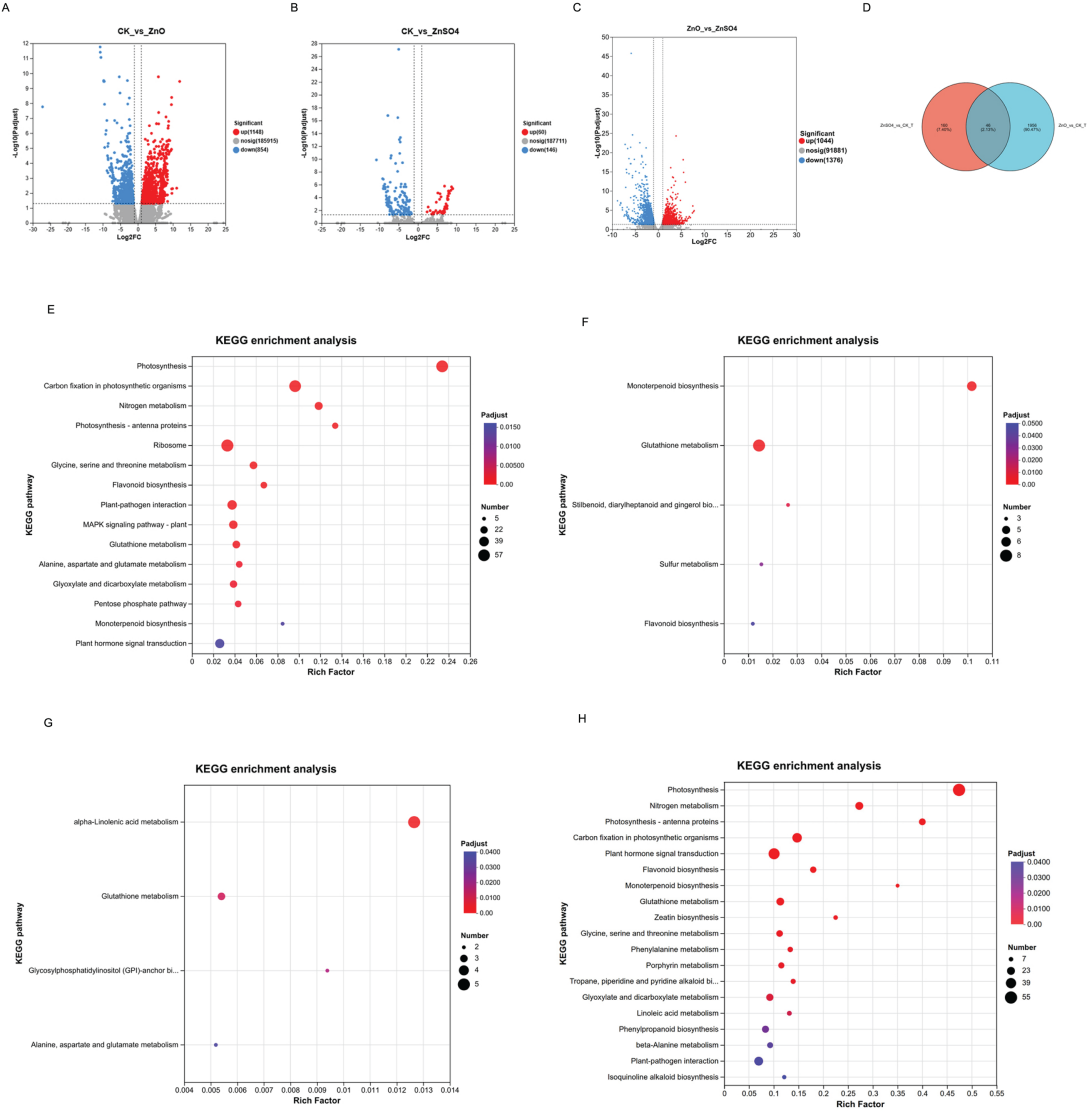
(A–H) Transcriptomic data analysis of *Lespedeza potaninii s*eeds.

Using |log2FC| ≥ 1 and FDR ≤ 0.05 as thresholds, we performed differential expression analyses for CK *vs*. ZnSO_4_, CK *vs*. ZnO-NPs, and ZnO-NPs *vs*. ZnSO4. In the CK_vs_ZnSO4 comparison, 206 transcripts were differentially expressed, of which 60 were upregulated and 146 were downregulated. In the CK_vs_ZnO-NPs comparison, 2,002 transcripts differed, with 1,148 upregulated and 854 downregulated. The ZnO-NPs *vs*. ZnSO_4_ comparison yielded 2,420 differentially expressed transcripts, including 1,044 upregulated and 1,376 downregulated (Figs. 5A–5C). A pairwise comparison of CK_vs_ZnSO4 and CK_vs_ZnO-NPs revealed 2,162 differentially expressed transcripts in total, 46 of which were common to both treatments (Fig. 5D).

Using a significance threshold of *P* < 0.05, pathway enrichment analysis identified the main biochemical routes associated with differentially expressed transcripts. In the ZnO-NPs_vs_CK comparison, transcripts were enriched in 116 metabolic pathways. The most significantly enriched pathways included photosynthesis; carbon fixation in photosynthetic organisms; nitrogen metabolism; photosynthesis-antenna proteins; glycine, serine, and threonine metabolism; flavonoid biosynthesis; glutathione metabolism; alanine, aspartate, and glutamate metabolism; glyoxylate and dicarboxylate metabolism; the pentose phosphate pathway; monoterpenoid biosynthesis; ribosome; plant–pathogen interaction; the MAPK signaling pathway—plants; and plant hormone signal transduction (Fig. 5E). In the ZnSO_4__vs_CK comparison, transcripts were enriched in 35 metabolic pathways. KEGG enrichment analysis identified eight pathways with significant overrepresentation, including monoterpenoid biosynthesis, glutathione metabolism, stilbenoid/diarylheptanoid/gingerol biosynthesis, sulfur metabolism, and flavonoid biosynthesis *(*Fig. 5F). KEGG analysis of transcripts shared by both ZnO-NPs and ZnSO_4_ seed-soaking treatments revealed significant enrichment in several pathways. Twelve DEGs were significantly enriched overall: five in α-linolenic acid metabolism, three in glutathione metabolism, two in glycosylphosphatidylinositol (GPI)-anchor biosynthesis, and two in alanine, aspartate, and glutamate metabolism (Fig. 5G). In the ZnO-NPs_vs_ZnSO_4_ comparison, transcripts were enriched in 119 metabolic pathways. The KEGG bubble chart highlighted 20 pathways with significant enrichment, among them Photosynthesis; Nitrogen metabolism; Photosynthesis—antenna proteins; Carbon fixation in photosynthetic organisms; Plant hormone signal transduction; Flavonoid biosynthesis; Monoterpenoid biosynthesis; Glutathione metabolism; Zeatin biosynthesis; Glycine, serine and threonine metabolism; Phenylalanine metabolism; Porphyrin metabolism; Tropane/piperidine/pyridine alkaloid biosynthesis; Glyoxylate and dicarboxylate metabolism; Linoleic acid metabolism; Phenylpropanoid biosynthesis; beta-Alanine metabolism; Plant-pathogen interaction; Isoquinoline alkaloid biosynthesis; and Tyrosine metabolism (Fig. 5H).

### Key pathways analysis

**Figure 6:**
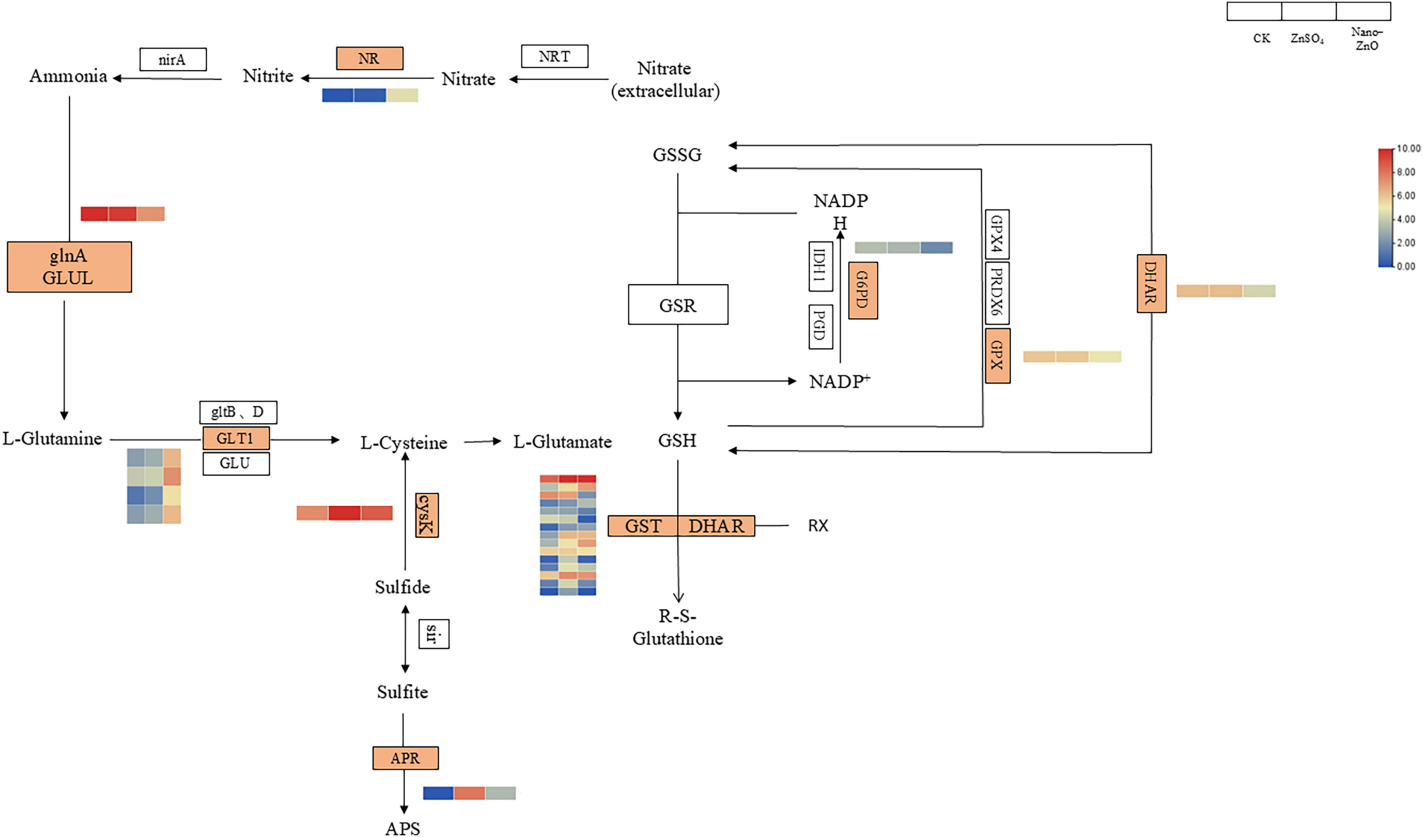
Nano-ZnO and ZnSO_4_ soaking treatment metabolism pathway analysis.

Analysis of gene expression levels related to the glutathione metabolism pathway revealed that, compared to the CK, the genes encoding *G6PD*, *DHAR*, *and GPX* were downregulated following both ZnO-NPs and ZnSO_4_ soaking treatments. Significant changes in gene expression levels were observed with the ZnO-NPs treatment. After ZnO-NPs seed soaking, nine out of 15 genes encoding *GST* were upregulated, and six were downregulated. Conversely, after ZnSO_4_ seed soaking, 12 out of 15 genes encoding *GST* were upregulated, and three were downregulated. These gene expression changes in the glutathione metabolism pathway were consistent with alterations in physiological indicators in seeds treated with ZnO-NPs and ZnSO_4_. Compared to the CK, genes encoding *NR* and *GLT1* were upregulated, while the gene encoding *glnA* was downregulated after ZnO-NPs and ZnSO_4_ soaking treatments. Notably, significant changes in gene expression levels were observed in the ZnO-NPs treatment but not in the ZnSO_4_ treatment. The ZnO-NPs treatment modulated nitrogen metabolism by regulating nitrite and glutamine levels, thereby enhancing seed drought resistance. Analysis of gene expression levels the sulfur metabolism pathway revealed that, compared to the CK, genes encoding *APR* and *cysK* were significantly upregulated following ZnO-NPs and ZnSO_4_ soaking treatments (Fig. 6).

## Discussion

Seed hardiness, marked by dense wax and cutin layers on the seed coat, impedes water, oxygen, and nutrient uptake and thereby reduces germination and preserves seed integrity (Carmen, John & Derrick, 2022). Although this barrier protects seeds, it also restricts germination and seedling establishment. Mechanical scarification with sandpaper has been shown to overcome dormancy and can raise germination to as high as 100% (Afza et al., 2023). Likewise, seed-priming treatments have improved physiological quality and mitigated hardiness-related dormancy in several okra (*Abelmoschus esculentus*) genotypes (Mekuria, 2023). In our study, scarification followed by soaking *Lespedeza potaninii* seeds in concentrated sulfuric acid for various durations increased germination to 63.34–97.56%. Soaking for 30 min produced the highest germination, so we used that pretreatment in subsequent experiments.

Under increasing abiotic stress, seed growth is progressively inhibited, but pre-sowing soaking treatments can reduce this damage. Studies of exogenous plant hormones show that in semi-arid regions, soaking maize (*Zea mays*) seeds in uniconazole raises abscisic acid (ABA) levels during grain filling while lowering gibberellin (GA) concentrations in the grains (Ahmad et al., 2018). A 10^−3^ mg/L solution of N-(Phenylmethyl)-9H-purin-6-amine (6-BA) improves both germination and physiological performance of maize seeds under drought (Yuan et al., 2014). Exogenous salicylic acid (SA) promotes tomato growth and photosynthetic efficiency by preserving stomatal function, maintaining redox balance, and supporting the antioxidant defense system (Mohd & Qazi, 2024). El-Mergawi & Abd El-Wahed (2020) reported that external SA and IAA increased endogenous SA and IAA levels in maize seeds, although they did not enhance germination. To identify effective soaking agents for *Lespedeza potaninii* under drought stress, we tested several agents at different concentrations and measured germination outcomes. Soaking with IAA and DA-6 produced no significant change in germination percentage under drought. In contrast, treatments with ZnO-NPs and ZnSO4 markedly improved seed vigor and germination capacity under the same stress conditions.

Seeds display physiological and biochemical responses to stress that reflect a limited tolerance range. Within this range, they adjust physiological parameters—for example, increasing antioxidant enzyme activities and osmolyte levels—to maintain homeostasis. When stress exceeds the tolerance threshold, these parameters decline and the seeds’ capacity to cope with stress diminishes (Rahmani et al., 2024). In our study, CAT activity, MDA content, SOD activity, and soluble sugar content did not differ significantly among seeds exposed to different soaking treatments. This finding suggests that the tested treatments did not increase ROS production or induce measurable cellular damage. By contrast, hydrogen peroxide (H_2_O_2_) content in *Lespedeza potaninii* seeds treated with ZnO-NPs and ZnSO_4_ was significantly lower than in the control group, a change that likely reflects reduced ROS generation and enhanced antioxidant defense. Previous work supports these observations. Yaghoubian et al. (2021) reported that zinc sulfate treatment raised chlorophyll, carotenoid, relative water content (RWC), and osmolyte levels in soybean seeds while lowering antioxidant enzyme activities. Similarly, Afzal Hussain (2018) found that ZnO-NPs decreased electrolyte leakage in wheat leaves, increased SOD and peroxidase (POD) activities, and alleviated chromium toxicity. In line with these reports, *Lespedeza potaninii* seeds treated with ZnO-NPs in our study exhibited higher glutathione reductase (GR) activity and reduced glutathione (GSH) content, lower glutathione peroxidase (GPX) activity and oxidized glutathione (GSSG) content, and a significantly increased GSH/GSSG ratio compared with the control. These shifts indicate an improved redox state and strengthened antioxidant capacity in response to ZnO-NPs treatment. Glutathione (GSH) is a central intracellular antioxidant that scavenges ROS and thereby limits lipid peroxidation. Plant-derived GSH also chelates heavy metal ions, forming complexes that alleviate metal-induced stress. Glutathione peroxidase (GPX) uses GSH to reduce H_2_O_2_ to GSSG (oxidized glutathione), which lowers both lipid peroxidation and H_2_O_2_ levels (Wang et al., 2023). In this study, ZnO-NPs treatment reduced GPX activity while increasing glutathione reductase (GR) activity in seeds, an effect that may stem from nano-ion action. The resulting decrease in GSH consumption and lower GSSG formation increased GSH content and helped preserve cellular integrity. Although both ZnO-NPs and ZnSO4 supply zinc ions, their distinct chemical structures produced different soaking effects. Both treatments enhanced seed germination under drought stress to some degree, but they produced different physiological responses, motivating further investigation by transcriptomic analysis.

**Figure 7:**
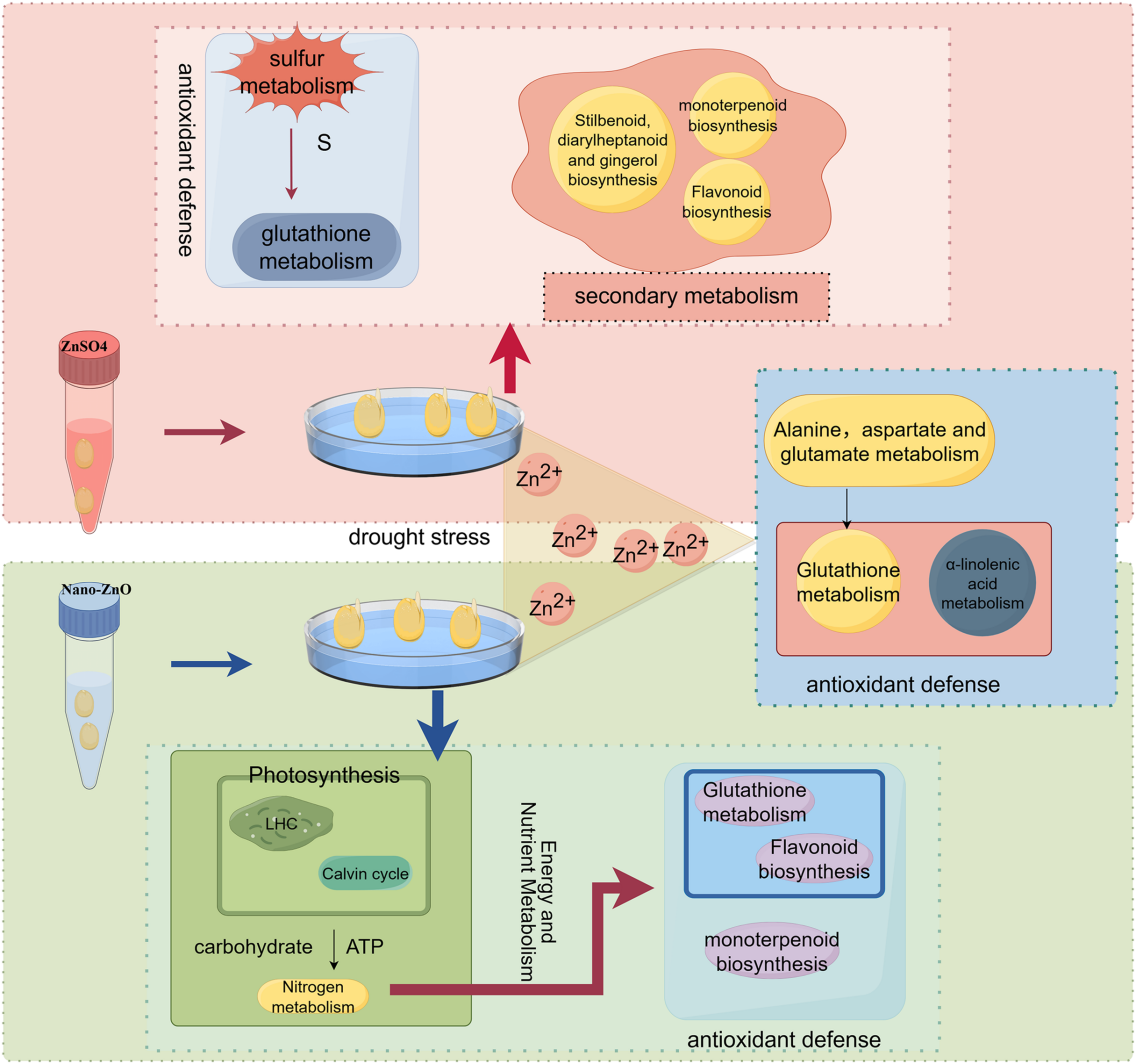
Mechanism of drought tolerance enhancement in *Lespedeza potaninii* seeds by Nano-ZnO and ZnSO_4_ seed soaking.

Nano ions enhance growth by improving physiological and biochemical processes, morphology, and nutrient uptake, which increases seed tolerance to abiotic stress and improves germination capacity. Nanoparticles can also modulate gene expression by altering specific miRNAs and by interacting with plants at the molecular level (Boykov, Shuford & Zhang, 2019; Ilona et al., 2020). In addition, they regulate tolerance-related genes and induce expression of genes involved in metal and oxidative stress responses (Kaveh et al., 2013). For example, zinc treatments raise ascorbic acid and S-hexyl-glutathione levels and thereby enhance the glutathione metabolic pathway. After 24 h of zinc ion exposure, relative mRNA levels of GR increased significantly, while GST and GPX levels decreased significantly; this pattern indicates increased production of reduced glutathione, which counteracts zinc-induced oxidative stress (Hong et al., 2022). Unique differentially expressed transcripts following ZnO-NPs soaking were associated with photosynthesis, nitrogen metabolism, multiple amino acid pathways (aspartate, alanine, glutamate, glycine, serine, threonine), flavonoid biosynthesis, and plant hormone signal transduction. Among these pathways, nitrogen metabolism is particularly important for drought tolerance because it modulates nitrate, nitrite, and glutamine levels that influence stress responses (Li et al., 2024). KEGG enrichment analysis of transcripts uniquely altered by ZnO-NPs soaking highlighted the nitrogen metabolism pathway. Regulation of seed nitrogen metabolism, especially *via* nitrate and glutamine, contributed to the observed improvement in drought tolerance. By contrast, transcripts uniquely affected by ZnSO_4_ soaking were predominantly enriched in sulfur metabolism and flavonoid biosynthesis. Inorganic sulfur serves as the primary source for synthesizing organic sulfur compounds in plants and microorganisms and is a structural element of sulfur-containing amino acids such as cysteine and methionine. Sulfur also contributes to membrane and cell wall components. Polypeptide thiols and the tripeptide glutathione participate in detoxifying harmful molecules and in intracellular redox regulation. Additionally, sulfur is a component of certain vitamins and coenzyme factors (Motohashi & Akaike, 2019). Consistent with these functions, KEGG enrichment after ZnSO_4_ soaking was strongly concentrated in the sulfur metabolism pathway; upregulation of APR and cysK expression therefore altered seed sulfur metabolism, mitigated metal ion–induced cellular damage, and enhanced seed drought tolerance. Sulfur ions introduced by ZnSO_4_ soaking likely mitigate drought stress by altering metabolic pathways that are active during water deficit. In contrast, ZnO-NPs likely mitigates drought effects by enhancing photosynthesis, interacting with the glutathione cycle, and scavenging reactive oxygen species, thereby preserving seed growth in a more reduced cellular environment. Both ZnO-NPs and ZnSO_4_ supply zinc ions, which boost antioxidant capacity and change ionic properties under abiotic stress (Neila et al., 2023). Hence, increasing zinc ion concentration within an appropriate range is expected to improve seed drought tolerance. In this study, co-expressed genes were predominantly enriched in α-linolenic acid metabolism, glutathione metabolism, glycosylphosphatidylinositol biosynthesis, and alanine, aspartate, and glutamate metabolism, indicating that zinc ions modulate drought tolerance mainly through these pathways. Notably, both ZnO-NPs and ZnSO_4_ soaking significantly enriched glutathione metabolism, and ZnO-NPs alone mapped 23 transcripts to this pathway. One of these transcripts encodes GPX and was markedly downregulated, consistent with physiological measurements showing reduced GPX activity. The simultaneous decline in GPX expression and activity likely explains the observed increase in GSH content in ZnO-NPs treated seeds (Quan et al., 2024). Transcriptomic data indicate that the differences between ZnO-NPs and ZnSO₄ treatments extend well beyond their modes of zinc ion supply. In the ZnO-NPs_vs_ZnSO_4_ comparison, pathways related to photosynthesis are specifically enriched, which suggests that nanoparticles may enter embryonic cells *via* physical adsorption or endocytosis and thereby directly modulate expression of chlorophyll biosynthesis genes and photosystem repair proteins (Rukhsar-Ul-Haq et al., 2023). This capacity for direct intervention allows ZnO-NPs to confer an early energetic advantage during seed germination. By contrast, ZnSO₄ lacks the surface effects and nanoscale penetrability of particles; its primary action therefore remains the slower uptake of ionic Zn^2+^. Moreover, divergent regulation of the nitrogen metabolism pathway further underscores the functional separation between the two treatments. ZnO-NPs treatment markedly upregulated NR and GLT1 expression, consistent with nano-zinc’s propensity to target and accumulate in meristems and thereby modulate key enzymes of nitrogen assimilation (Li et al., 2022). In contrast, ZnSO₄ exerted a weaker effect on nitrogen metabolism but significantly enriched the sulfur metabolic pathway, suggesting that sulfate ions are preferentially absorbed and mobilized during seed water uptake and swelling and are diverted to the synthesis of sulfur-containing defense compounds. This metabolic shunt toward sulfur plant’s antioxidant defense system that following ZnO-NPs under severe drought (Fig. 7).

## Conclusion

Seed soaking with 0.10 mg/L ZnO-NPs and 0.04% ZnSO_4_ were effective in enhancing drought resistance of *Lespedeza potaninii* during germination. ZnO-NPs primarily enhances energy and nutrient metabolism (particularly nitrogen metabolism). This enhancement bolsters the plant’s antioxidant defense system (including glutathione metabolism), enabling effective scavenging of ROS and thereby improving drought resistance. ZnSO_4_ seed soaking supplies sulfur for glutathione synthesis, promoting the glutathione cycle and enhancing antioxidant capacity. Both treatments likely involve zinc ions enhancing antioxidant synthesis and cell membrane stability by regulating antioxidant defense and amino acid metabolism, particularly glutathione and α-linolenic acid metabolism. This coordination helps mitigate oxidative damage, aiding in drought tolerance.

## Supplemental Information

10.7717/peerj.21054/supp-1 Supplemental Information 1 Effects of different seed hardiness Breaking treatments on the germination percentage of *Lespedeza potaninii*.

10.7717/peerj.21054/supp-2 Supplemental Information 2 Effects of different concentrations of seed soaking agents on the germination indicators of *Lespedeza potaninii*.

10.7717/peerj.21054/supp-3 Supplemental Information 3 Effects of different seed soaking agents on germination indices of *Lespedeza potaninii* under drought stress.

10.7717/peerj.21054/supp-4 Supplemental Information 4 Effects of different seed soaking agents on oxidase, secondary metabolites and osmotic adjustment substances of *Lespedeza potaninii*.

10.7717/peerj.21054/supp-5 Supplemental Information 5 Characterization of ZnO-NPS-fig.s1.

10.7717/peerj.21054/supp-6 Supplemental Information 6 Germination phenotype of 0.1mg/L nano-ZnO soaked *Lespedeza potaninii* seeds under sorbitol-simulated drought stress.

10.7717/peerj.21054/supp-7 Supplemental Information 7 Germination phenotype of 0.04% ZnSO₄-soaked *Lespedeza potaninii* seeds under sorbitol-simulated drought stress.

10.7717/peerj.21054/supp-8 Supplemental Information 8 Germination phenotype of water soaked *Lespedeza potaninii* seeds under sorbitol-simulated drought stress.

## Additional Information and Declarations

### Data Availability

The following information was supplied regarding data availability:

The raw sequences are available at NCBI Sequence Read Archive: PRJNA1095388.

## References

[ref-1] Abhilash A, Dayal A, Thomas N, Sharan A, Vipul V (2023). Effect of zinc oxide (ZnO) nanoparticles on the storability of onion (*Allium cepa* L.) seeds under ambient condition. International Journal of Plant & Soil Science.

[ref-2] Afza H, Palupi ER, Ilyas S, Herlina L (2023). Evaluation of hard seed in Indonesia Local Mungbean (*Vigna radiata* L.). IOP Conference Series: Earth and Environmental Science.

[ref-3] Afzal Hussain SAMR (2018). Zinc oxide nanoparticles alter the wheat physiological response and reduce the cadmium uptake by plants. Environmental Pollution.

[ref-4] Ahmad I, Kamran M, Ali S, Cai T, Bilegjargal B, Liu T, Han Q (2018). Seed filling in maize and hormones crosstalk regulated by exogenous application of uniconazole in semiarid regions. Environmental Science and Pollution Research.

[ref-5] Aoyama L, Cook EJ, Hallett LM (2023). Intraspecific variation in native grass seedling plastic trait response to water stress depends on the context of annual grass invasion. Restoration Ecology.

[ref-6] Bardgett Richard D, Bullock James MBJ, Sandra L, Peter M, Urs S, Nicholas O, Mathilde C, Giselda D, Ellen LF, David JM, Le Provost Gaëtane LJ, Shan L, Kenny P, Mahesh S, Xiangyang H, Huakun Z, Li M, Weibo R, Xiliang L, Yong D, Yuanheng L, Hongxiao S (2021). Combatting global grassland degradation. Nature Reviews Earth & Environment.

[ref-7] Bhattacharjee B, Ali A, Rangappa K, Choudhury BU, Mishra VK (2023). A detailed study on genetic diversity, antioxidant machinery, and expression profile of drought-responsive genes in rice genotypes exposed to artificial osmotic stress. Scientific Reports.

[ref-8] Bourhim MR, Cheto S, Qaddoury A, Hirich A, Ghoulam C (2022). Chemical seed priming with zinc sulfate improves quinoa tolerance to salinity at germination stage. Environmental Sciences Proceedings.

[ref-9] Boykov IN, Shuford E, Zhang B (2019). Nanoparticle titanium dioxide affects the growth and microRNA expression of switchgrass (*Panicum virgatum*). Genomics.

[ref-10] Carmen PT, John GH, Derrick JM (2022). Time of sowing and cultivar effects on hardseededness and germination of subterranean clover seeds. New Zealand Journal of Agricultural Research.

[ref-11] Chen L, Ma P, Li J, Liu J, Guo F, Wang Y, Zhang J (2024). *Lespedeza potaninii* Vass seed yield response to plant density and phosphate fertilization in Northwest China. European Journal of Agronomy.

[ref-12] Du Y, Zhao Q, Chen L, Yao X, Zhang H, Wu J, Xie F (2020). Effect of drought stress during soybean R2-R6 growth stages on sucrose metabolism in leaf and seed. International Journal of Molecular Sciences.

[ref-13] Duraisamy K, Amirthalingam M, Govindhan T, Kim J, Hasegawa K, Palanisamy S (2022). Fabrication of zinc oxide nanorods using plant latex serum as a green matrix for the sustainable management of root-knot nematodes. Materials Letters.

[ref-14] El-Mergawi RA, Abd El-Wahed MSA (2020). Effect of exogenous salicylic acid or indole acetic acid on their endogenous levels, germination, and growth in maize. Bulletin of the National Research Centre.

[ref-15] Garstecka Z, Antoszewski M, Adamska AM, Krauklis D, Niedojadło K, Kaliska B, Hrynkiewicz K, Dąbrowska GB (2023). *Trichoderma viride* colonizes the roots of *Brassica napus* L., alters the expression of stress-responsive genes, and increases the yield of canola under field conditions during drought. International Journal of Molecular Sciences.

[ref-16] Hong ZZ, Cheng LZ, Han L, Kai GQ, Xi WC, Zhen CJ, Xing LA (2022). Glutathione metabolism in *Cryptocaryon irritans* involved in defense against oxidative stress induced by zinc ions. Parasites & Vectors.

[ref-50] Huang W, He Y, Zhao X, Wang H, Zhu Y (2023a). Effects of warming and precipitation reduction on physiological characters of dominant psammophytes seeds in Horqin sandy land, northeast China. Acta Physiologiae Plantarum.

[ref-52] Huang X, Rao G, Peng X, Xue Y, Hu H, Feng N, Zheng D (2023b). Effect of plant growth regulators DA-6 and COS on drought tolerance of pineapple through bromelain and oxidative stress. BMC Plant Biology.

[ref-17] Ilona P, Inese K, Anastasija P, Marija J, Vjačeslavs G, Marina K (2020). The impact of zinc oxide nanoparticles on cytotoxicity, genotoxicity, and miRNA expression in barley (*Hordeum vulgare* L.) seedlings. The Scientific World Journal.

[ref-19] Jing W, Aihong Z, Shunyu X, Changyun L, Haoran P, Yuxia W, Xiaozhou M, Haitao C, Mao R, Xianchao S (2022). Transcriptome analysis reveals the mechanism of zinc ion-mediated plant resistance to TMV in *Nicotiana benthamiana*. Pesticide Biochemistry and Physiology.

[ref-20] Karim MR, Zhang Y, Zhao R, Chen X, Zhang F, Zou C (2012). Alleviation of drought stress in winter wheat by late foliar application of zinc, boron, and manganese. Journal of Plant Nutrition and Soil Science.

[ref-21] Kaveh R, Li Y, Ranjbar S, Tehrani R, Brueck CL, Van Aken B (2013). Changes in *Arabidopsis thaliana* gene expression in response to silver nanoparticles and silver ions. Environmental Science & Technology.

[ref-22] Kimura K, Kono A, Yamada S, Koyanagi TF, Okuro T (2022). Grazing and heat stress protection of native grass by a sand-fixing shrub in the arid lands of northern China. Journal of Arid Land.

[ref-23] Lal SA, Sushmita S, Vidya C, Bhai PC, Koushik C, Lokesh KT, Kumar MM (2023). Zinc-sulphate and Zn-EDTA enhances Zn and other nutrients and yield and quality of table-purpose peanut cultivars. Communications on Soil Science and Plant Analysis.

[ref-24] Li G, Guo X, Sun W, Hou L, Wang G, Tian R, Wang X, Qu C, Zhao C (2024). Nitrogen application in pod zone improves yield and quality of two peanut cultivars by modulating nitrogen accumulation and metabolism. BMC Plant Biology.

[ref-25] Li Y, Zhang L, Yu Y, Zeng H, Deng L, Zhu L, Chen G, Wang Y (2022). Melatonin-induced resilience strategies against the damaging impacts of drought stress in rice. Agronomy.

[ref-45] Lin T, Chen X, Ren Y, Qing B, Zhang M, Mo Z, Wang S (2024). Effects of iron oxide nanocoatings on the seed germination, seedling growth, and antioxidant response of aromatic rice grown in the presence of different concentrations of rice straw extracts. Journal of Nanoparticle Research.

[ref-26] Lu Y, Liu H, Chen Y, Zhang L, Kudusi K, Song J (2022). Effects of drought and salt stress on seed germination of ephemeral plants in desert of northwest China. Frontiers in Ecology and Evolution.

[ref-27] Mapodzeke JM, Adil MF, Sehar S, Karim MF, Saddique MAB, Ouyang Y, Shamsi IH (2021). Myriad of physio-genetic factors determining the fate of plant under zinc nutrient management. Environmental and Experimental Botany.

[ref-28] Mekuria BS (2023). Effect of seed priming methods on seed quality of okra (*Abelmoschus esculentus* (L.) Moench) genotypes. Advances in Agriculture.

[ref-29] MingXu Z, LingYu Z, YuanYuan H, JinPeng H, GuoWen H, Ying Z, Aziz K, YouCai X, JinLin Z (2023). Potential roles of iron nanomaterials in enhancing growth and nitrogen fixation and modulating rhizomicrobiome in alfalfa (*Medicago sativa* L.). Bioresource Technology.

[ref-30] Mohd S, Qazi F (2024). Exogenous application of salicylic acid via seed soaking improved growth and photosynthetic efficiency by maintaining stomatal organisation, redox homeostasis, and antioxidant defense system in tomato (*Solanum lycopersicum* L.). Acta Physiologiae Plantarum.

[ref-31] Montanha GS, Rodrigues ES, Marques JPR, de Almeida E, Colzato M, Pereira De Carvalho HW (2020). Zinc nanocoated seeds: an alternative to boost soybean seed germination and seedling development. SN Applied Sciences.

[ref-32] Motohashi H, Akaike T (2019). Sulfur-utilizing cytoprotection and energy metabolism. Current Opinion in Physiology.

[ref-33] Muhammad WM, Muhammad I, Iqbal H, Abida P, Khizar HB, Muhammad A, Sumaira T, Muhammad A, Mehwish M, Tauqeer S, Khursheed M, Nazim N (2022). Seed nano-priming with Zinc Oxide nanoparticles in rice mitigates drought and enhances agronomic profile. PLOS ONE.

[ref-34] Mustafa H, Ilyas N, Akhtar N, Raja NI, Zainab T, Shah T, Ahmad A, Ahmad P (2021). Biosynthesis and characterization of titanium dioxide nanoparticles and its effects along with calcium phosphate on physicochemical attributes of wheat under drought stress. Ecotoxicology and Environmental Safety.

[ref-35] Neila A, Angeline VB, Chrisna S, Maryke L (2023). Zn fertilizer and mycorrhizal inoculation effect on bread wheat cultivar grown under water deficit. Life.

[ref-36] Quan W, Zhao T, Du Z, Fan J, Kang Y, Xue W (2024). Effect of gravity-induced pressure treatment on the growth and development of mung bean sprouts: Integrated analysis of widely targeted metabolomics and transcriptomics. Food Bioscience.

[ref-37] Rahmani F, Sodaeizadeh H, Biouki RY, Ardakani MAH, Aliabadi KK (2024). Effect of bio-priming on morphological, physiological and essential oil of chamomile (*Matricaria chamomilla* L.) under salinity stress. South African Journal of Botany.

[ref-38] Rajesh K, Abhishek D, Mamta D, Lakshika S, Mohan SM (2024). Stimulatory effect of ZnO nanoparticles as a nanofertilizer in seed priming of pearl millet (*Pennisetum glaucum*) and their bioactivity studies. South African Journal of Botany.

[ref-39] Reddy KJ, Mehera B, Kumar P (2023). Effect of biofertilizers and foliar application of zinc on growthand yield of groundnut (*Arachis hypogea* L.). International Journal of Plant & Soil Science.

[ref-40] Rukhsar-Ul-Haq, Kausar A, Hussain S, Javed T, Zafar S, Anwar S, Hussain S, Zahra N, Saqib M (2023). Zinc oxide nanoparticles as potential hallmarks for enhancing drought stress tolerance in wheat seedlings. Plant Physiology and Biochemistry.

[ref-41] Sarkar MM, Sarkar A, Roy S (2024). Fertigation of NaCl-stressed lentil and soybean plants with silica nanoparticles improves seed yield and nutritional attributes. Plant Nano Biology.

[ref-42] Sharaf-Eldin MA, Alshallash KS, Alharbi KR, Alqahtani MM, Etman AA, Yassin AM, Azab ES, El-Okkiah SAF (2022). Influence of seed soaking and foliar application using ozonated water on two sweet pepper hybrids under cold stress. Sustainability.

[ref-43] Shuang S, Huo X, Chen Q, Dai R, Li J, Yan J, Jiang X, Tan Y, Zhang Z (2025). Exogenous methyl jasmonate-mediated physiological and transcriptomic network improves thrips tolerance in alfalfa (*Medicago* sativa). Journal of Pest Science.

[ref-44] Tang W, Sun J, Yu X, Zhou F, Liu S, Liu M, Lu Y, Yang Y (2022). Fenclorim increasing butachlor selectivity between wheat and *Roegneria kamoji* by seed soaking. Agronomy.

[ref-46] Umair Hassan M, Aamer M, Umer Chattha M, Haiying T, Shahzad B, Barbanti L, Nawaz M, Rasheed A, Afzal A, Liu Y, Guoqin H (2020). The critical role of zinc in plants facing the drought stress. Agriculture.

[ref-47] Van Nguyen D, Nguyen HM, Le NT, Nguyen KH, Nguyen HT, Le HM, Nguyen AT, Dinh NTT, Hoang SA, Van Ha C (2022). Copper nanoparticle application enhances plant growth and grain yield in maize under drought stress conditions. Journal of Plant Growth Regulation.

[ref-48] Wang J, Wu H, Wang Y, Ye W, Kong X, Yin Z (2024). Small particles, big effects: how nanoparticles can enhance plant growth in favorable and harsh conditions. Journal of Integrative Plant Biology.

[ref-49] Wang Y, Xing M, Gao X, Wu M, Liu F, Sun L, Zhang P, Duan M, Fan W, Xu J (2023). Physiological and transcriptomic analyses reveal that phytohormone pathways and glutathione metabolism are involved in the arsenite toxicity response in tomatoes. Science of the Total Environment.

[ref-51] Wu Z, Chang P, Zhao J, Li D, Wang W, Cui X, Li M (2022). Physiological and transcriptional responses of seed germination to moderate drought in *Apocynum venetum*. Frontiers in Ecology and Evolution.

[ref-53] Yang B, Chen S, Zheng Z, Zeng J, Liu J, Zhao H, Zheng Y (2024). Genome-wide association studies for rice seed germination under drought stress using 3VmrMLM. Food and Energy Security.

[ref-18] Yaghoubian I, Ghassemi S, Nazari M, Raei Y, Smith DL (2021). Response of physiological traits, antioxidant enzymes and nutrient uptake of soybean to *Azotobacter Chroococcum* and zinc sulfate under salinity. South African Journal of Botany.

[ref-54] Yuan Z, Wang C, Li S, Li X, Tai F (2014). Effects of different plant hormones or PEG seed soaking on maize resistance to drought stress. Canadian Journal of Plant Science.

[ref-55] Zhang M, He S, Qin B, Jin X, Wang M, Ren C, Cao L, Zhang Y (2020a). Exogenous melatonin reduces the inhibitory effect of osmotic stress on antioxidant properties and cell ultrastructure at germination stage of soybean. PLOS ONE.

[ref-56] Zhang Y, Li Y, Hassan MJ, Li Z, Peng Y (2020b). Indole-3-acetic acid improves drought tolerance of white clover via activating auxin, abscisic acid and jasmonic acid related genes and inhibiting senescence genes. BMC Plant Biology.

[ref-57] Zhu Z, Li Y, Liu T, Shi R, Xu X, Song Z, Wang Y (2024). Comparison of the differences in tolerance to drought stress across FiveClematisSpecies based on seed germination and seedling growth. Horticulturae.

