# Supplementary figures and images for: Zn-based seed priming enhances drought resistance during germination of Lespedeza potaninii: physiological and transcriptomic insights
Source: PeerJ. 2026 Apr 7;14:e21054. doi: 10.7717/peerj.21054 (PMC13068015; doi:10.7717/peerj.21054)

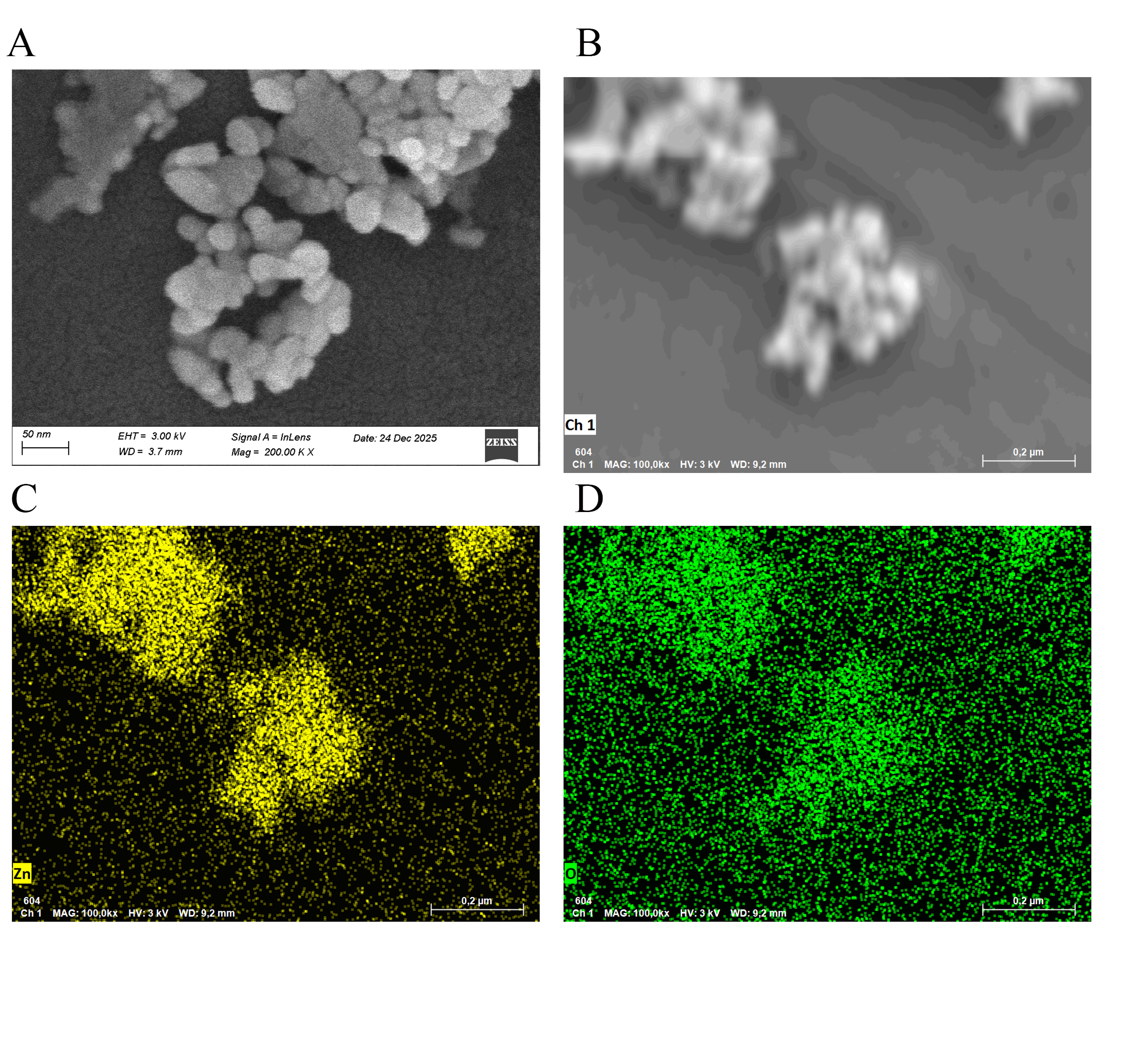

Supplement: Supplemental Information 5 [file peerj-14-21054-s005.png]

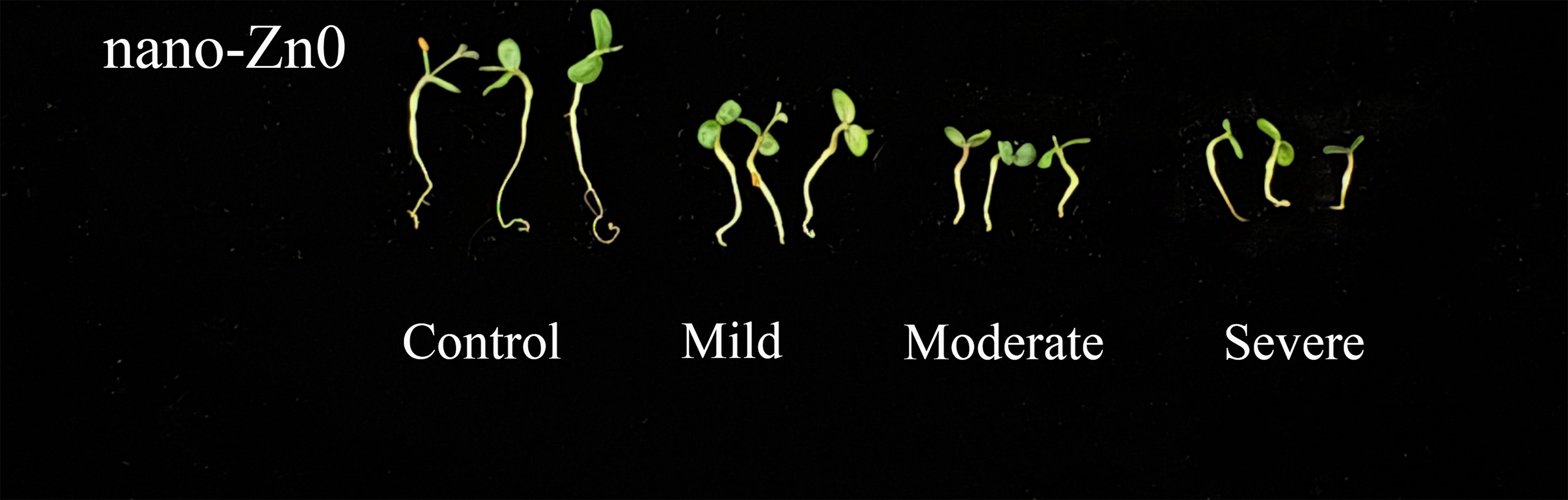

Supplement: Supplemental Information 6 [file peerj-14-21054-s006.png]

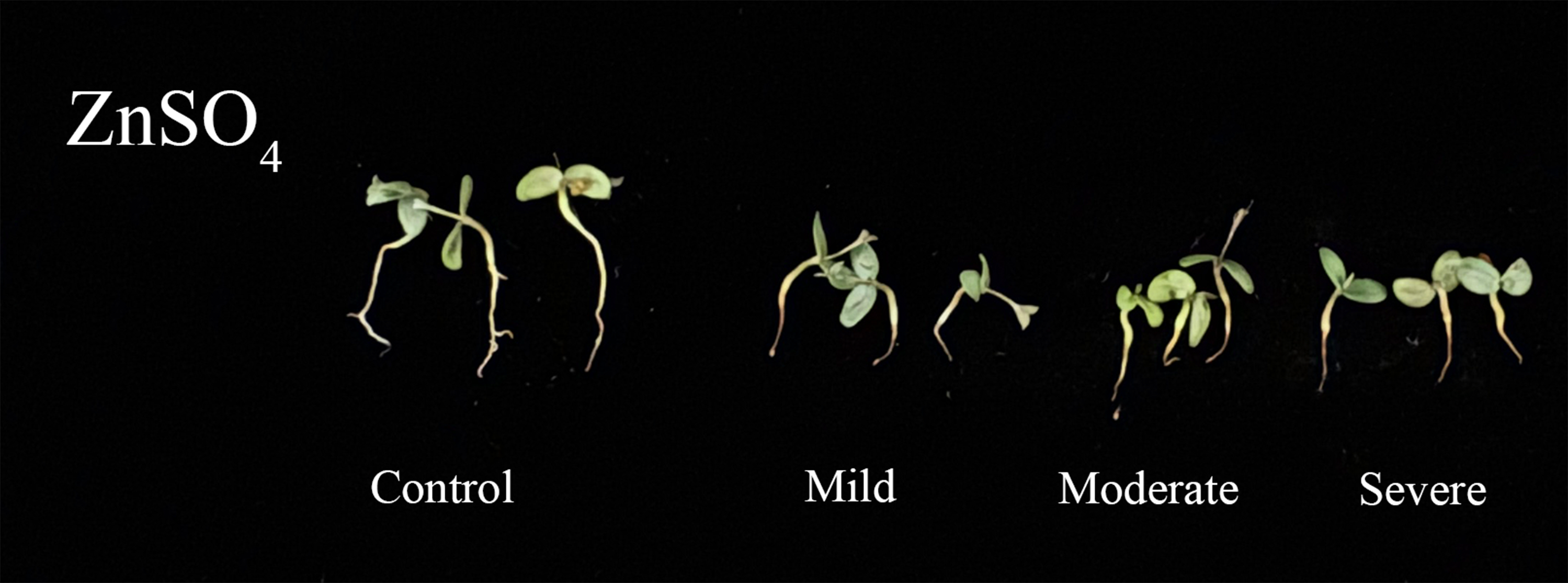

Supplement: Supplemental Information 7 [file peerj-14-21054-s007.png]

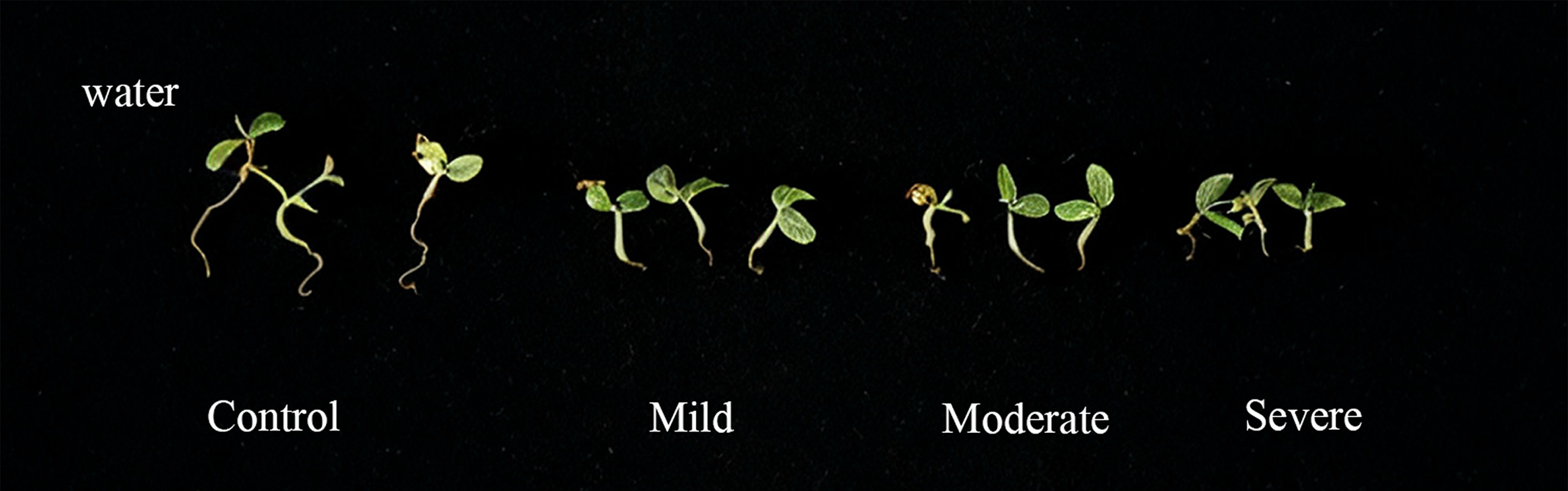

Supplement: Supplemental Information 8 [file peerj-14-21054-s008.png]
